# Influence of Reward Motivation on Directed Forgetting in Younger and Older Adults

**DOI:** 10.3389/fpsyg.2020.01764

**Published:** 2020-07-31

**Authors:** Holly J. Bowen, Sara N. Gallant, Diane H. Moon

**Affiliations:** ^1^Department of Psychology, Southern Methodist University, Dallas, TX, United States; ^2^Leonard Davis School of Gerontology, University of Southern California, Los Angeles, CA, United States

**Keywords:** directed forgetting, reward anticipation, aging, memory, reward motivation

## Abstract

An important feature of the memory system is the ability to forget, but aging is associated with declines in the ability to intentionally forget potentially due to declines in cognitive control. Despite cognitive deficits, older adults are sensitive to affective manipulations, such as reward motivation, and reward anticipation can improve older adults’ memory performance. The goal of the current studies was to examine the effect of reward motivation on directed remembering and forgetting. Participants were healthy CloudResearch/Turk Prime workers aged 18–35 and 60–85. In Experiment 1, we conducted a typical item-method directed forgetting task using neutral words presented one at a time followed by a to-be-remembered (TBR) or to-be-forgotten (TBF) cue. A recognition memory test followed that included all words from the encoding task, as well as new words. We replicated prior findings of better memory for TBR compared to TBF items, but not typical age-related differences in recognition of TBF items. In Experiments 2–4, we repeated this paradigm except that in the second block of trials, each word was presented with a high ($0.75) or low ($0.01) reward cue indicating the value that could be earned if the item was successfully Remembered or Forgotten (depending on cue). During recognition, correct responses to target items (both TBR and TBF) resulted in the associated reward, but incorrect “old” responses resulted in a loss of $0.50. In three experiments, high rewards led to better memory for younger and older adults compared to low rewards, regardless of the directed cue to remember or forget the word. In Experiments 3 and 4, older adults showed typical deficits in directed forgetting, but this was across reward conditions. For older adults, there was no evidence that including reward motivation improved cognitive control abilities as high value reward anticipation did not improve directed forgetting. Instead, in line with hypotheses, high compared to low value reward anticipation leads to engagement of processes that result in better memory regardless of the TBR or TBF cue, and reward anticipation bolsters memory in a relatively automatic, rather than strategic, fashion that overrides one’s ability to cognitively control encoding processes.

## Introduction

An important and adaptive feature of the memory system is the ability to forget irrelevant or unwanted information. Forgetting can occur *unintentionally*, due to decay of a memory trace, but there are also circumstances that necessitate *intentional* forgetting—for example, to avoid interference with similar or overlapping information, to update incorrect or missing information in memory with new information, or as an emotion regulation strategy for memories that evoke negative affect. To study intentional forgetting in the lab, directed forgetting paradigms indicate to participants that some stimuli are to-be-remembered (TBR) and other stimuli are to-be-forgotten (TBF) via cues presented after each stimulus presentation (MacLeod, 1998). Aging is associated with well-documented increases in unintentional forgetting (Maylor, 1993), but when older adults are directed to intentionally forget information, they often have difficulty doing so compared to younger adults (Zacks et al., 1996; Titz and Verhaeghen, 2010). The cognitive and neural mechanisms responsible for the directed forgetting effect are hotly debated (Anderson and Hanslmayr, 2014; Aguirre et al., 2017), but a prevailing hypothesis concerning older adults’ paradoxical forgetting abilities is that age-related declines in cognitive control lead to reductions in goal-directed memory processes and the inability to inhibit unwanted information which leads to continued encoding of items they have been instructed to forget (Sahakyan et al., 2008; Titz and Verhaeghen, 2010; Gallant et al., 2018). In other words, to intentionally forget, one must engage inhibitory cognitive control and resist goal-irrelevant TBF stimuli, but as we age, the ability to inhibit attention to distracting or unnecessary information declines, thereby leading older adults to remember TBF items to a greater extent than younger adults. Neuroimaging evidence provides additional support for this hypothesis as reduced intentional forgetting in older adults is associated with reduced engagement of frontal lobe inhibitory control regions (Rizio and Dennis, 2014).

While executive function and other cognitive processes are associated with an age-related decline (Salthouse, 2010; Murman, 2015; Salthouse, 2019), affective functioning, such as sensitivity to rewards, is relatively preserved or maintained in healthy aging (Harada et al., 2013; Mather, 2016). In several contexts, older and younger adults show similar activation in the reward network to gain and loss anticipation (Samanez-Larkin et al., 2007; Spaniol et al., 2015; Geddes et al., 2018; Bowen et al., 2020) and gain and loss feedback (Bowen et al., 2019), but valence differences have also been reported in striatal regions that respond robustly to rewarding outcomes compared to loss outcomes (Samanez-Larkin et al., 2007, 2014; Schott et al., 2007; Vink et al., 2015). Memory performance has been shown to be enhanced by high compared to low reward anticipation (Castel et al., 2002; Castel, 2007; Spaniol et al., 2014; Cohen et al., 2016; Bowen et al., 2020), as well as positive feedback (Eppinger et al., 2011; Mather and Schoeke, 2011), in younger as well as older adults.

Reward can enhance older adults’ ability to remember, so the critical question in this set of studies is whether it could also improve directed forgetting. One interpretation of the memory findings reported above is that even in older adults who suffer cognitive deficits, intact reward anticipation increases cognitive control over episodic memory formation. When motivated by a reward cue, cognitive control processes are engaged to successfully remember the high- compared to low-value items to a greater extent (see Cohen et al., 2014; Eich and Castel, 2016, for a discussion of this). Neuroimaging evidence supports this idea that reward motivation increases cognitive control due to projections between the ventral tegmental area of the reward network to the prefrontal cortex during reward processing (for a review, see Ferdinand and Czernochowski, 2018). Since cognitive control is thought to underlie older adults’ reduced ability to intentionally forget, rewards could potentially increase goal-directed remembering *and* forgetting. A second interpretation for the motivated memory findings above is that reward motivation enhances processing of high-value compared to low-value stimuli, but this processing is relatively automatic, rather than controlled (e.g., Cohen et al., 2019; Bowen et al., 2020). Neuroimaging provides evidence for this interpretation as reward anticipation boosts activation in the ventral tegmental area and triggers dopaminergic modulation of hippocampal consolidation processes. Presenting reward cues during stimulus presentation (i.e., before remember/forget instructions) may make forgetting even more difficult due to the relatively automatic cascade of processes within and between the ventral tegmental area and hippocampus during reward anticipation (e.g., Adcock et al., 2006; Bowen et al., 2020). Furthermore, reward anticipation has been shown to increase semantic processing of word stimuli—which are typically employed as stimuli in directed forgetting paradigms—in particular when a high reward is at stake. This results in elaborative encoding and increased memory for high- compared to low-value information (Cohen et al., 2016).

Considering reported age-related impairments in directed forgetting (Titz and Verhaeghen, 2010), it is important to determine whether older adults’ ability to intentionally forget could be improved by extrinsic motivation via monetary reward, like it has been shown to increase remembering of high-compared to low-value information. A few prior studies have demonstrated that reward motivation does influence the directed forgetting effect in younger adults. In an effort to empirically test the possibility that participants’ lack of motivation to search and recover TBF items may actually be driving the directed forgetting effect, Macleod (1999) offered participants a reward ($0.50) for any additional TBF words they could recall after an initial recall test for all TBR and TBF items. Despite this added motivation, participants reported very few additional TBF words during the second recall task, suggesting that the directed forgetting effect may not be driven by differential withholding of recovered TBF words. Macleod (1999) implemented reward motivation during the retrieval phase, so it is unclear from these results whether reward anticipation could influence cognitive control processes engaged during the encoding phase of the directed forgetting task. To answer this question, Friedman and Castel (2011) used a directed forgetting task where remember and forget cues were replaced with numerical values and participants were told to try and maximize their points with the following instructions: Words followed by +5, if recalled, would result in a gain of 5 points, but words followed by −5, if recalled, would result in a loss of 5 points, effectively making these words TBR and TBF, respectively. The authors found a stronger directed forgetting effect in the motivation block compared to a baseline block with no motivation manipulation. Finally, in a recent study, instead of replacing remember/forget cues with reward values, Ren et al. (2018) orthogonalized remembering/forgetting and reward/loss, by presenting TBR and TRF cues along with reward and loss cues, after presentation of each stimulus during encoding. The reward cues indicated how many points would be rewarded for successful remembering and forgetting on the subsequent recognition task as well as how many points would be lost for unsuccessful remembering and forgetting. They found that words associated with rewards led to a typical directed forgetting effect with better memory for TBR items compared to TBF items, but the threat of losses made it difficult for participants to forget, and there was no significant difference in recognition between TBR and TBF words.

### The Current Studies

The goal of the current set of studies was to examine the effect of reward anticipation on age differences in directed forgetting in healthy younger and older adults. Hypotheses and a power analysis were preregistered on the Open Science Framework^1^. In Experiment 1, we wanted to establish directed forgetting effects in an online sample of younger and older adults recruited from CloudResearch/Turk Prime (Litman et al., 2017). This first study was done using a typical item-method directed forgetting task with neutral words without any motivational incentives. Based on prior research, we expected an age-related decline in the overall directed forgetting effect (i.e., the difference between memory for TBR vs. TBF words). In Experiment 2, we tested the effect of high-and low-value motivational incentives (monetary rewards) on directed forgetting in younger and older adults. Based on research described above, we suspected that high rewards would increase memory for TBR items compared to low or no reward in all participants. Compared to younger adults, we predicted that high rewards would also reduce the directed forgetting effect compared to a baseline condition of no reward in older adults, making TBF words even harder to forget. In Experiment 3, we investigated participant strategy during the recognition task, specifically whether they intentionally withheld their memory of TBF words in order to receive a reward. Experiment 3 followed the same procedure as Experiment 2, but after the recognition task, participants were offered an additional reward for each TBF word that they could freely recall to determine if they were intentionally withholding their memory for TBF words. While we did not have age-related hypotheses about this third experiment, given Macleod’s (1999) findings, we hypothesized that participants would freely recall very few TBF items, which would be indicative of a cognitive strategy employed during encoding to modulate remembering and forgetting abilities, rather than a motivational strategy on the part of the participant to increase earnings. Finally, Experiment 4 followed the same procedures as Experiment 3 with the exception that participants were asked multiple choice questions during instructions to ensure that they understood the reward contingencies. We expected the findings from Experiment 3 would replicate, indicating the effects were reliable.

## Experiment 1

While an age-related decline in directed forgetting has been shown (Titz and Verhaeghen, 2010), this has yet to be established in an online sample of young and older adults. Although participants recruited through crowdsourcing platforms like Amazon’s Mechanical Turk (MTurk) are diverse, they do not necessarily represent the general population, which may reflect that Internet users typically differ from non-Internet users in systematic ways (Paolacci and Chandler, 2014). For example, there is some evidence that MTurk workers tend to be more educated, underemployed, more liberal, less extraverted, and more socially anxious than the general population (Shapiro et al., 2013). Given these potential differences between online and lab-based samples, the goal of Experiment 1 was to determine baseline directed forgetting effects in an online sample of young and older adults. During the study, participants completed an item directed forgetting task for neutral words. We predicted that young adults would show a larger directed forgetting effect than older adults.

### Method

#### Participants

A power analysis using G^∗^Power (Faul et al., 2007) indicated that a sample size of *n* = 48 in each group would provide 95% power to detect a within–between interaction effect of η_p_^2^ = 0.08 with α = 0.05. The effect size η_p_^2^ = 0.08 is a conservative estimate (60%) of an effect size η_p_^2^ = 0.14 reported from an Age × Reward × Recognition interaction in a study with a similar design (Spaniol et al., 2014). We chose this conservative estimate to deal with potentially inflated effect sizes due to underpowered samples in the prior work. In Experiment 1, the final sample after exclusions included 50 young adults ranging in age from 22 to 29 years (*M* = 26.18, *SD* = 2.21) and 51 older adults ranging in age from 60 to 77 years (*M* = 65.37, *SD* = 4.49). All participants were recruited via CloudResearch/Turk Prime (Litman et al., 2017) and located in the United States. Participants were compensated $5 USD for approximately 45 min of participation. All participants provided informed consent in accordance with procedures approved by the Institutional Review Board at Southern Methodist University.

To ensure data quality, participants were required to complete attention checks that were dispersed throughout the survey to make sure they were reading task instructions (Oppenheimer et al., 2009). This included multiple choice questions in which specific responses were required such as, “Please select option three” and “What was this experiment about?,” with the options “Current events,” “Marketing,” “Products,” and “Other.” The instructions for the latter question told participants to select “Other” and type the word “Silver” in the response box (e.g., Gallant et al., in press; Mather et al., 2012). At the end of the task, participants were also asked to indicate whether they wrote down any of the words during encoding to help their performance on the memory task. If participants did not pass these attention checks or indicated that they had written down words, their data were excluded from analysis. Data were also excluded if participants learned English after the age of 7, had fewer than 12 years of education (to better match in-lab samples of older adults who typically have at least some post-secondary education), indicated a diagnosis of a psychiatric and/or neurological disorder, or were taking psychoactive medications. Based on these criteria, nine young adults and 11 older adults were excluded from analyses.

Characteristics of the final sample are displayed in Table 1. Older adults had more years of education, *t*(97) = 2.14, *p* = 0.04, and scored higher on the Shipley Vocabulary test, *t*(99) = 3.51, *p* = 0.001. These are age differences that are commonly reported in the literature (e.g., Gallant and Yang, 2014; Gallant et al., 2018). Older adults had lower rates of anxiety, *t*(99) = 2.13, *p* = 0.04, but there were no age differences in depression or stress, *ts* ≤ 1.55, *ps* ≥ 0.13; based on Depression, Anxiety, and Stress Scale-21 items (DASS-21) scoring, these scores were all within the “Normal” range (Lovibond and Lovibond, 1995). We also sought to characterize age differences in motivational behavior using the Behavioral Inhibition System/Behavioral Activation System (BIS/BAS) scales (Carver and White, 1994), which is theorized to be composed of separate behavioral inhibition and activation systems. Relative to young adults, older adults showed lower levels of behavioral inhibition, *t*(99) = 2.31, *p* = 0.023. In terms of activation, older adults had lower levels of drive, *t*(99) = 2.01, *p* = 0.04, and fun seeking than young adults, *t*(97) = 2.71, *p* = 0.008, but—importantly—they did not differ in reward responsiveness, *t* = 0.29, *p* = 0.77.

**TABLE 1:** Sample characteristics.

	Experiment 1 (*n* = 101)		Experiment 2 (*n* = 96)		Experiment 3 (*n* = 95)		Experiment 4 (*n* = 85)	
				
	Younger adults	Older adults	Younger adults	Older adults	Younger adults	Older adults	Young adults	Older adults

Characteristic	*M (SD)*	*M (SD)*	*M (SD)*	*M (SD)*	*M (SD)*	*M (SD)*	*M (SD)*	*M (SD)*
Age	26.18 (2.21)	65.37 (4.49)	26.02 (2.37)	65.81 (3.93)	25.69 (2.90)	65.09 (5.14)	25.87 (3.55)	65.69 (5.05)
Years of Education	14.84 (2.08)	*15.97 (3.09)	15.33 (1.69)	15.02 (2.28)	15.45 (1.60)	15.47 (2.57)	14.90 (2.12)	15.66 (2.48)
Shipley	32.80 (3.28)	**35.12 (3.36)	32.31 (3.85)	**35.67 (2.89)	31.58 (5.40)	**34.47 (3.90)	31.80 (4.25)	**35.38 (3.36)
BAS Drive	*11.60 (2.43)	10.65 (2.33)	*11.04 (2.45)	9.77 (2.61)	**11.74 (2.57)	9.68 (2.18)	11.44 (2.64)	10.40 (2.69)
BAS FS	*11.49 (2.07)	10.36 (2.08)	*11.13 (2.27)	9.83 (2.81)	**11.57 (2.32)	10.23 (2.36)	10.95 (2.35)	9.96 (2.55)
BAS RR	16.82 (2.07)	16.94 (1.99)	16.60 (2.52)	16.28 (2.51)	17.17 (2.11)	16.45 (2.10)	16.75 (2.32)	17.04 (2.32)
BIS	*21.24 (4.79)	19.10 (4.54)	*20.89 (5.43)	18.49 (4.96)	^*b*^ −	^*b*^ −	20.25 (5.55)	19.78 (3.79)
DASS: Anxiety^a^	*3.14 (3.77)	1.76 (2.64)	**3.08 (3.71)	0.77 (1.52)	**3.06 (3.46)	0.89 (1.40)	**4.42 (4.68)	1.44 (1.97)
DASS: Depression	3.98 (5.21)	2.61 (3.83)	**5.00 (5.86)	2.23 (3.67)	*4.06 (4.87)	1.96 (3.86)	**6.05 (5.92)	2.22 (2.83)
DASS: Stress	4.38 (4.57)	3.14 (3.42)	**5.15 (4.67)	2.60 (3.21)	**5.19 (4.34)	1.80 (1.85)	**6.38 (5.34)	2.56 (3.05)

	***n (%)***	***n (%)***	***n (%)***	***n (%)***	***n (%)***	***n (%)***	***n (%)***	***n (%)***

**Sex**								
Female	27 (54)	37 (72.5)	27 (56.3)	34 (70.8)	28 (58.3)	30 (66.7)	19 (47.5)	26 (57.8)
Male	23 (46)	14 (27.4)	21 (43.8)	14 (29.2)	20 (41.7)	14 (31.1)	21 (52.5)	18 (40)
**Ethnicity**								
Hispanic	4 (8)	0 (0)	5 (10.4)	0 (0)	5 (10.4)	3 (6.7)	4 (10)	0 (0)
Not Hispanic	46 (92)	51 (100)	43 (89.6)	48 (100)	42 (87.5)	40 (88.9)	36 (90)	44 (97.8)
**Racial Group**								
African American	7 (14)	2 (3.9)	3 (6.3)	0 (0)	5 (10.4)	3 (6.7)	9 (22.5)	4 (8.9)
American Indian	0 (0)	0 (0)	1 (2.1)	0 (0)	0 (0)	1 (2.2)	0 (0)	0 (0)
Asian/Pacific Islander	5 (10)	1 (1.9)	5 (10.4)	0 (0)	3 (6.3)	0 (0)	4 (10)	0 (0)
Caucasian	37 (74)	47 (92)	36 (75)	48 (100)	35 (72.9)	40 (88.9)	26 (65)	41 (91.1)
Other	1 (2.0)	0 (0)	3 (6.3)	0 (0)	5 (10.4)	1 (2.2)	1 (2.5)	0 (0)

BAS FS, Behavioral Activation System (BAS) Fun-Seeking subscale; BAS RR, BAS Reward-Responsivity subscale; BIS, Behavioral Inhibition System subscale; DASS, Depression, Anxiety, and Stress Scale (21-item). *This age group had a significantly higher score than the other age group within experiments, at *p* < 0.05; ** at *p* < 0.01. ^*a*^Older adults in Experiment 1 vs. Experiment 2 had significantly higher mean anxiety scores, *p* = 0.04. There were no other significant group differences in sample characteristics across experiments. ^*b*^A programming error led to omission of BIS scores in Qualtrics for Experiment 3.

#### Materials

The experiment was programmed and run using Qualtrics survey software (Qualtrics, Provo, UT, United States). A total of 90 neutral nouns were selected from the word list provided by Janschewitz (2008) and split into three lists of 30. During the item directed forgetting task, participants saw 60 words, evenly split across two encoding blocks followed by a recognition task that was composed of the 60 old words and 30 new words as lures. As such, the three stimuli lists were counterbalanced as old words that were presented across the two encoding blocks and lure words presented only during the recognition task. Each of the old word lists were further randomly split into two sets of 15 words, which were each paired with a TBR cue (RRRR) or a TBF cue (FFFF).

Three questionnaires were administered at the end of the experiment. The Shipley Institute of Living Vocabulary task (Shipley, 1940) measures crystallized intelligence with a 40-item vocabulary test. The BIS/BAS (Carver and White, 1994) is a 24-item self-report questionnaire designed to measure the complementary motivational systems. The DASS-21 (Lovibond and Lovibond, 1995) assesses emotional states of depression, anxiety, and stress.

#### Procedure

Participants first completed the item directed forgetting task. They were told that the purpose of the study was to understand their ability to selectively prioritize and remember some words over others. Participants were instructed to study a series of words for a later memory task, some of which would be followed by the cue “RRRR,” which meant they should remember the word, or the cue “FFFF,” which meant they should forget the word. Participants first completed six practice trials to familiarize themselves with encoding. Each trial began with a fixation cross in the center of the screen for 500 ms, followed by a word for 1,500 ms. To discourage participants from writing down the words, they were asked to use the mouse to check a box located directly below the word once they had finished studying it. After the word, a blank screen as an interstimulus interval (ISI) appeared for 500 ms, immediately followed by either the RRRR or FFFF cue for 1,000 ms. During encoding, words were presented in a pseudo-randomized order across two blocks of 30 trials, which each included 15 TBR and 15 TBF words. There was a 30-s break between blocks.

Following encoding, participants completed a non-verbal distractor task for approximately 5 min. The recognition task followed during which participants saw a series of 90 words (60 old words intermixed with 30 lures) and were instructed to indicate whether the word was old, meaning they previously studied it regardless of the cue it was associated with, or new, meaning they did not study it. Responses were self-paced and made via a mouse click. After recognition, participants completed the questionnaires and demographic information.

### Results

**FIGURE 1:**
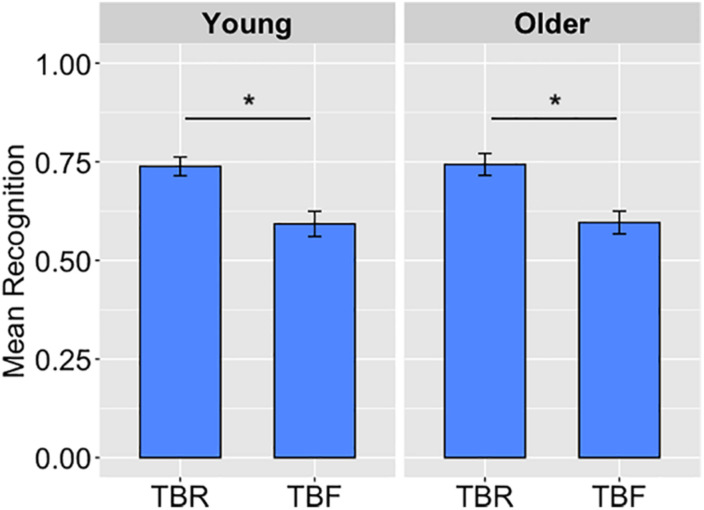
The average hits to recognize to-be-remembered (TBR) and to-be-forgotten (TBF) words in Experiment 1, displayed as a function of age group, illustrate a main effect of cue (*p* < 0.001) in which both age groups showed better recognition of TBR than TBF words. Error bars represent standard error of the mean. ^∗^*p* < 0.001.

Results were analyzed using SPSS and interpreted in terms of statistical significance (α = 0.05) and effect size using partial eta square (η_p_^2^). Bonferroni-corrected pairwise comparisons were modeled into the analyses. Data were visualized using ggplot2 (Wickham, 2016) in R Studio. To determine how recognition was affected by the memory cue, we analyzed hits to correctly recognize words as old as well as false alarms to incorrectly classify new words as old as a measure of guessing. Average hit rates were submitted to a 2 (Age Group: Young, Older) × 2 (Cue: TBR, TBF) repeated-measures ANOVA (Figure 1), with age as the only between-subjects factor. A main effect of cue, *F*(1,99) = 35.53, *p* < 0.001, η_p_^2^ = 0.26, showed that hit rates were higher for TBR words (*M* = 0.74, *SD* = 0.18) than for TBF words (*M* = 0.59, *SD* = 0.22; Figure 1). The main effect of age and the interaction was not significant, *Fs* ≤ 0.02, *ps* ≥ 0.89.

An independent-samples *t-*test on average false alarm rates across age groups showed that young adults had higher false alarm rates (*M* = 0.22, *SD* = 0.18) than older adults (*M* = 0.09, *SD* = 0.12), *t*(99) = 3.54, *p* < 0.001.

### Discussion

In Experiment 1, both age groups were similar in their ability to remember TBR words and intentionally forget TBF words, which suggests there may be no age difference in directed forgetting when young and older adults are sampled from online crowdsourcing platforms. This contradicts our hypothesis as well as the findings of previous studies (Zacks et al., 1996; Sahakyan et al., 2008; Gallant et al., 2018) and meta-analyses (Titz and Verhaeghen, 2010) that have demonstrated smaller directed forgetting effects in older relative to young adults in laboratory-based tasks.

One possible explanation for age-equivalent finding is that older adults using CloudResearch/Turk Prime have higher cognitive abilities (e.g., greater cognitive reserve, more computer savvy, more motivated to seek out opportunities) than the average older adult participating in laboratory studies. Consistent with this notion, there is evidence that digital literacy (e.g., Internet and e-mail use) may positively impact cognitive performance in older adults by reducing cognitive decline (Xavier et al., 2014; Klimova, 2016). However, young adults in the current study showed a lower hit rate for TBR words (*M* = 0.73) when compared to previous investigations (*M* = 0.87 in Collette et al., 2014; *M* = 0.89 in Gallant et al., 2018) and also showed a higher false alarm rate than older adults, an age difference that is typically reversed (e.g., Huh et al., 2006). This finding might imply that our young adults were not fully attending to the encoding task and, as a result, did not encode the words as well as older adults. Prior work has shown that, relative to lab-based participants, young adults completing studies via MTurk are more likely to be distracted by other activities such as using their cell phone, watching television, browsing the Internet, or talking with friends (Clifford and Jerit, 2014). By dividing their attention among other tasks, young adults from online settings may be inadvertently reducing their cognitive performance.

In the second experiment, we investigated the effect of reward motivation on young and older adults’ directed forgetting performance. However, prior to implementing reward incentives, we had participants complete a baseline item directed forgetting block with no rewards to see if we could replicate the age-equivalent directed forgetting effect observed in Experiment 1.

## Experiment 2

In Experiment 2, we modified the procedure of Experiment 1 to include high and low rewards for memory performance. During encoding, participants first completed a no-reward block to establish baseline directed forgetting. In the second block, each stimulus was paired with either a high ($0.75) or low reward ($0.01) prior to the memory cue, which indicated how much money could be earned if TBR words were successfully remembered or TBF words were successfully forgotten. We expected that high rewards would increase memory for TBR words compared to low or no reward in all participants. We also predicted that high rewards would reduce older, but not younger, adults’ directed forgetting effect relative to a no-reward baseline condition by making TBF words even harder to forget.

### Method

#### Participants

Based on the exclusion criteria used in Experiment 1, 24 young adults and 26 older adults were excluded from analyses. The final sample after exclusions included 48 young adults ranging in age from 19 to 29 years (*M* = 26.02, *SD* = 2.38; 27 females) and 48 older adults ranging in age from 60 to 75 years (*M* = 65.81, *SD* = 3.93; 34 females). All participants were recruited via CloudResearch/Turk Prime (Litman et al., 2017) and provided informed consent for their participation. Participants were compensated $4 USD for approximately 45 min of work in addition to the incentives they received based on their memory performance.

The final sample characteristics are displayed in Table 1. There was no age difference in education, *t* = 0.76, *p* = 0.45, but older adults scored higher on the Shipley vocabulary test than young adults, *t*(94) = 4.82, *p* < 0.001. Relative to young adults, older adults had lower levels of depression, *t*(94) = 2.78, *p* = 0.007, anxiety, *t*(94) = 3.99, *p* < 0.001, and stress, *t*(94) = 3.11, *p* = 0.002; all scores fell in the “Normal” range. On the BIS/BAS, older adults showed lower behavioral inhibition than young adults, *t*(91) = 2.18, *p* = 0.03, as well as lower total activation, *t*(91) = 2.11, *p* = 0.04, including lower drive, *t*(92) = 2.35, *p* = 0.02, and fun seeking, *t*(92) = 2.42, *p* = 0.02. Age groups did not differ in reward responsiveness, *t*(91) = 0.46, *p* = 0.65.

#### Materials

The experiment was programmed and run using Qualtrics survey software (Qualtrics, Provo, UT, United States). A total of 120 neutral nouns were selected from the word list provided by Janschewitz (2008) and split into four lists of 30 words. During the directed forgetting task of Experiment 2, participants again completed two encoding blocks, which included a no-reward block of 30 words, followed by a reward block that included 30 words paired with a high reward intermixed with 30 words that were paired with a low reward. During the recognition task, participants viewed 90 old words intermixed with 30 new lure words. The four word lists were counterbalanced such that they equally served as no reward, high reward, low reward, and new words across participants. Each list of 30 words was further randomly split into two subsets of 15 words, which were each paired with a TBR or TBF cue.

#### Procedure

The directed forgetting task followed the same procedure as Experiment 1, except that participants first completed an encoding block with no rewards, followed by an encoding block in which words were equally paired with high- or low-reward values. Participants were not informed that they could receive a reward for their performance until the second block. This was done to ensure that knowledge of monetary incentives did not influence performance on the no-reward block, which provided a baseline measure of directed forgetting. Each trial of the no-reward block proceeded the same as in Experiment 1. Participants completed eight practice trials followed by the no-reward block, which included 15 TBR words intermixed with 15 TBF words. After a 30-s break, participants started the second block, in which each word was presented with a monetary cue indicating the reward that they could earn if the word was successfully remembered or forgotten. Reward-block trials proceeded the same as no-reward trials, except that each word was paired with a reward, either $0.75 or $0.01, that appeared directly above the word. To differentiate rewards, high rewards appeared in green colored font (RGB decimal: 50, 205, 50), whereas low rewards appeared in blue colored font (RGB decimal: 52, 152, 219). Reward-block trials included 15 high-reward TBR words, 15 high-reward TBF words, 15 low-reward TBR words, and 15 low-reward TBF words.

After encoding, participants completed a non-verbal filler task for 5 min followed by a recognition task for the 90 old words intermixed with 30 new lures. They were told to indicate whether each word was old or new and that the reward for each word they correctly identified as old would be based on the monetary cue ($0.75 or $0.01) it was associated with during encoding. To discourage participants from committing a false alarm to new words to increase their reward, they were told they would lose $0.50 for each new word incorrectly identified as old. The recognition task followed the same procedure as that of Experiment 1, after which participants completed the Shipley Vocabulary Task, BIS/BAS, DASS-21, and a demographic questionnaire.

After the experiment, rewards were calculated based on performance and administered to participants’ CloudResearch/Turk Prime account. Rewards for TBR words were calculated based on the total number of words that were successfully remembered (i.e., identified as old). In contrast, rewards for TBF words were calculated based on the total number of words that were successfully forgotten (i.e., identified as new). The false alarm penalty was calculated based on the total number of new words recognized as old and was subtracted from their overall reward.

### Results

**FIGURE 2:**
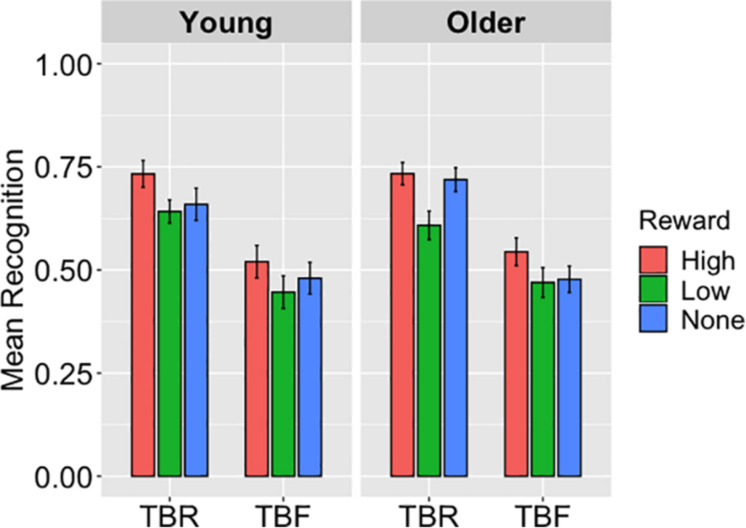
The average hits for to-be-remembered (TBR) and to-be-forgotten (TBF) words in Experiment 2, displayed as a function reward magnitude and age group, illustrate a main effect of cue, with more TBR words recognized than TBF words in both age groups (*p* < 0.001). A main effect of reward (*p* < 0.001) is also apparent, with high-reward words recognized more than low- and no-reward words. Error bars represent standard error of the mean.

Recognition performance is displayed in Figure 2. Average hit rates were submitted to a 2 (Age Group: Young, Older) × 3 (Reward: No Reward, High Reward, Low Reward) × 2 (Cue: TBR, TBF) ANOVA. A main effect of cue, *F*(1,94) = 81.33, *p* < 0.001, η_p_^2^ = 0.46, showed that hits were higher for TBR (*M* = 0.68, *SD* = 0.17) than for TBF words (*M* = 0.49, *SD* = 0.22). There was also a main effect of reward, *F*(2,188) = 11.96, *p* < 0.001, η_p_^2^ = 0.11. Pairwise comparisons showed that high-reward words (*M* = 0.73, *SD* = 0.21) were better recognized than low-reward words (*M* = 0.63, *SD* = 0.22, *p* < 0.001) and no-reward words (*M* = 0.69, *SD* = 0.24, *p* = 0.04). The difference between recognition of low-reward and no-reward words was not significant (*p* = 0.08). There were no other significant main effects or interactions in the ANOVA, *Fs* ≤ 2.19, *ps* ≥ 0.11.

Age differences in false alarms to incorrectly identify new words as old were also analyzed. The *t*-test showed that older adults made fewer false alarms (*M* = 0.13, *SD* = 0.14) than young adults (*M* = 0.22. *SD* = 0.20), *t*(94) = 2.39, *p* = 0.02.

### Discussion

Like Experiment 1, young and older adults in Experiment 2 were similar in their overall directed forgetting performance, recognition was higher for TBR than TBF words, and older adults again showed a tendency for fewer false alarms. As mentioned previously, one possibility is that online samples of young adults are dividing their attention among other tasks (Clifford and Jerit, 2014), reducing their ability to pay full attention during encoding. As such, in Experiment 3, we repeated the procedure of Experiment 2, but we added an additional task to the encoding phase that required participants’ attention. Following each cue, participants were required to indicate whether an arrow cue (presented as “<” or “>”) was pointing to the left or right side of the screen. This task was intended to be simple enough to keep participants’ attention engaged, but to not detract from the cognitive processes required to intentionally remember and forget words.

Experiment 2 also partially supported our hypotheses as recognition was better for high-reward than for low- or no-reward words, but this did not vary as a function of whether words were cued as TBR or TBF. With regard to the effect of reward on directed forgetting, one possibility is participants are intentionally withholding their memory of high- and low-reward TBF words in order to maximize their overall payout. This would imply a motivational explanation for participants’ forgetting rates as opposed to a process-based explanation in which participants are using cognitive resources to limit encoding of TBF words (Macleod, 1999). A second goal of Experiment 3 was therefore to better understand participant strategy during the motivated directed forgetting task.

## Experiment 3

The purpose of Experiment 3 was two-fold: first, to increase participant engagement during encoding and, second, to further investigate the effect of rewards on participants’ memory for TBF words. We modified the directed forgetting task by including a simple arrow-detection task following the presentation of each memory cue during encoding. We also implemented a surprise recall task for TBF words, modeled after Macleod (1999). Specifically, following an initial memory task, participants were offered an additional reward for every TBF word that they could freely recall. If participants show better recall of high- and low-reward TBF words relative to no-reward TBF words, this might imply that they were intentionally withholding their memory of TBF words to receive a higher payout.

### Method

#### Participants

Based on the exclusion criteria described in Experiments 1 and 2, 23 young adults and 13 older adults were excluded from analyses. The final sample after exclusions included 49 young adults ranging in age from 18 to 31 years (*M* = 25.69, *SD* = 2.90; 28 females, one unidentified sex) and 46 older adults ranging in age from 59 to 79 years (*M* = 65.09, *SD* = 5.14; 30 female, three unidentified sex). All participants were recruited via CloudResearch/Turk Prime (Litman et al., 2017) and provided informed consent for their participation. Similar to Experiment 2, participants were compensated $4 USD for approximately 45 min of work in addition to the incentives they received based on their memory performance during the recognition and recall task.

Characteristics of the final sample are displayed in Table 1. Older adults scored higher than young adults on the Shipley Vocabulary test, *t*(93) = 2.88, *p* = 0.005, but age groups did not differ in total years of education, *t* = 0.05, *p* = 0.96. Older adults showed lower levels of depression, *t*(93) = 2.29, *p* = 0.02, anxiety, *t*(93) = 3.84, *p* < 0.001, and stress, *t*(93) = 4.96, *p* < 0.001, than young adults, but all scores fell within the “Normal” range. On the BIS/BAS, older adults scored lower on overall behavioral activation, *t*(87) = 3.51, *p* = 0.001, including drive, *t*(89) = 4.12, *p* < 0.001, and fun seeking, *t*(88) = 2.72, *p* = 0.008; there was no age difference in responsiveness to reward, *t* = 1.62, *p* = 0.11. Due to a programming error, one item from the behavioral inhibition scale was not presented to participants and so it was not possible to compute this score for either age group.

#### Procedure

Experiment 3 used the same materials and protocol as Experiment 2 with a few modifications. Each encoding trial followed the same procedure except that after the memory cue (RRRR or FFFF), an arrow (< or >) appeared in the center of the screen and participants were required to indicate the direction of the arrow via button press. This was done to ensure that participants stayed engaged with the task and to discourage them from selectively writing down TBR words. Following encoding and a 5-min filler task, the same recognition test as Experiment 2 was administered. After recognition, participants were told they could earn an additional $0.10 for each TBF word that they could recall. They were invited to recall any words that they could remember, including TBR words, but they were only rewarded for recall of TBF words. They had 1.5 min to type their responses into a response box. After the recall task, participants completed the same questionnaires and tasks as Experiments 1 and 2. Their rewards were calculated based on memory performance and administered to their CloudResearch/Turk Prime account.

### Results

**FIGURE 3:**
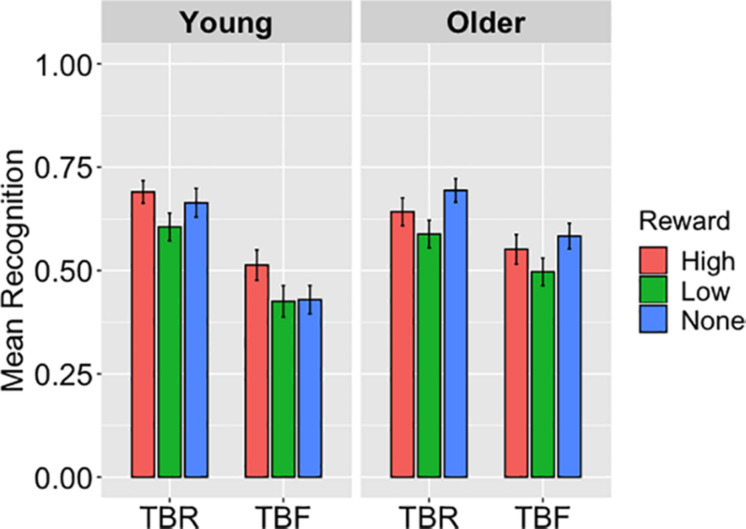
The average hits for to-be-remembered (TBR) and to-be-forgotten (TBF) words in Experiment 3, displayed as a function of reward magnitude and age group, illustrate a main effect of cue (*p* < 0.001) with better recognition of TBR than TBF words as well as an effect of reward (*ps* < 0.01), such that low-reward words were recognized to lesser extent than both high- and no-reward words. The age-by-cue interaction is also apparent as older adults recognized more TBF words than young adults (*p* = 0.04), but the same proportion of TBR words. The figure further shows the age-by-reward interaction in which older adults had superior recognition of no-reward words versus young adults (*p* = 0.01) but similar recognition of high- and low-reward words. Error bars represent standard error of the mean.

Recognition performance is displayed in Figure 3. Average hit rates were entered to a 2 (Age Group: Young, Older) × 3 (Reward: High, Low, None) × 2 (Cue: TBR, TBF) repeated-measures ANOVA. This analysis showed a main effect of reward, *F*(2,186) = 9.04, *p* < 0.001, η_p_^2^ = 0.09. According to pairwise comparisons, hit rates were reduced for low-reward words (*M* = 0.53, *SD* = 0.21) relative to high-reward (*M* = 0.60, *SD* = 0.20; *p* < 0.001) and no-reward words (*M* = 0.59, *SD* = 0.19; *p* = 0.009); hit rates for high-reward words did not differ from no-reward words (*p* > 0.99). A main effect of cue, *F*(1,93) = 69.54, *p* < 0.001, η_p_^2^ = 0.42, also showed that hit rates were higher for TBR words (*M* = 0.65, *SD* = 0.17) than for TBF words (*M* = 0.50, *SD* = 0.21). The main effect of age was not significant, *F* = 1.25, *p* = 0.26.

There was a significant two-way interaction of age group and cue, *F*(1,93) = 6.68, *p* = 0.01, η_p_^2^ = 0.07. Pairwise comparisons showed that older adults recognized more TBF words (*M* = 0.55, *SD* = 0.19) than young adults (*M* = 0.46, *SD* = 0.22; *p* = 0.04), suggesting they were less able to intentionally forget TBF words than their young counterparts. Hits for TBR words did not differ, *p* = 0.75. The age group-by-reward interaction was also significant, *F*(2,186) = 2.94, *p* = 0.05, η_p_^2^ = 0.03, such that recognition of no-reward words was higher in older adults (*M* = 0.64, *SD* = 0.17) than young adults (*M* = 0.55, *SD* = 0.20), *p* = 0.01; there was no age difference between the other reward conditions. The remaining interactions were not significant, *Fs* ≤ 1.31, *ps* ≥ 0.27.

The *t-*test on false alarm rates across age groups showed no difference between young (*M* = 0.21, *SD* = 0.18) and older adults (*M* = 0.25, *SD* = 0.20), *t* = 1.05, *p* = *0.29*.

Proportional recall rates were analyzed in a 2 (Age Group: Young, Older) × 3 (Reward: High, Low, None) × 2 (Cue: TBR, TBF) ANOVA. In general, recall was low (*M* = 0.04, *SD* = 0.04). There was a marginal main effect of cue, *F*(1,93) = 3.51, *p* = 0.06, η_p_^2^ = 0.04, which showed that recall rates were higher for TBR (*M* = 0.04, *SD* = 0.06) than for TBF words (*M* = 0.03, *SD* = 0.04). The remaining effects and interactions were not significant, *Fs* ≤ 1.11, *ps* ≥ 0.33.

### Discussion

The results of Experiment 3 revealed an age-related difference in the directed forgetting effect consistent with prior in-lab experiments (Sahakyan et al., 2008; Titz and Verhaeghen, 2010; Gallant et al., 2018). Older adults had more difficulty cognitively controlling their memory and therefore recognized more TBF items compared to younger adults, but recognition of TBR items did not differ between age groups. Unlike Experiments 1 and 2, younger adults did not commit more false alarms than older adults. We believe that age differences in directed forgetting, but no age differences in false alarm rates, emerged in this experiment, and not in Experiment 1 or 2, because of the arrow task that was added to increase participant engagement, particularly for younger adults. As noted, when doing online versus in-lab experiments, young adults often divide their attention, potentially reducing their ability to pay attention to the task and key instructions, like remember or forget cues. This inclusion of the arrow task seems to have increased younger adults’ ability to intentionally forget which is driving the age-related interaction. One concern was that adding this task would reduce performance overall, especially for older adults, but across all three experiments, the recognition rates for both age groups are relatively consistent. Like Experiment 2, high-reward led to better memory than low-reward items, but in Experiment 3, there were no differences between high-reward and no-reward trials. This boost for no-reward trials in Experiment 3 may also be related to greater task engagement particularly in the first block of trials when there were fewer competing trials and less memory interference.

Finally, replicating the findings from Experiments 1 and 2, recognition was better for TBR compared to TBF words. We added the surprise rewarded recall task after recognition to understand whether this was strategic. Participants may have intentionally withheld their memory for TBF words in order to maximize their overall payout, indicative of a motivational retrieval strategy rather than a process-based explanation in which participants are limiting encoding of TBF words. The recall task was based on the design from Macleod (1999) and in agreement their results, we found that participants freely recalled very few words, but did recall slightly more TBR than TBF words, providing evidence against the motivational prediction that participants were withholding their memory at the time of retrieval.

## Experiment 4

The results of Experiments 2 and 3 imply that high rewards do not enhance the ability to intentionally forget TBF words and, instead, enhance overall remembering. Given that these experiments were conducted online without an experimenter present to explain instructions, it is possible that participants may have misunderstood how rewards would be administered for TBF words. Specifically, participants may have thought that rewards were only associated with remembering *in general*, thus assuming they would forgo $0.75 if they forgot TBF words. This could explain why high rewards had a general effect on overall memory rather than a differential effect on remembering or forgetting (a reward-by-cue interaction). To rule out this possibility, in Experiment 4, we repeated Experiment 3 and added comprehension questions during the instructions to ensure that participants understood how rewards would be administered for TBR and TBF trials. We also sought to replicate the age-by-cue interaction that we observed in Experiment 3 when we added the arrow detection task to increase participant engagement.

### Method

#### Participants

The same exclusion criteria from Experiments 1–3 were applied to Experiment 4, resulting in the exclusion of 31 young adults and 19 older adults from analyses. The final sample after exclusions included 40 young adults ranging from 18 to 30 years of age (*M* = 25,88, *SD* = 3.55; 19 females) and 45 older adults ranging from 61 to 89 years of age (*M* = 65.59, *SD* = 5.06; 26 females, one unidentified sex). All participants were located in the United States, recruited from CloudResearch/Turk Prime (Litman et al., 2017), and provided informed consent for their participation. They were compensated $4 USD for approximately 45 min of work plus the incentives received based on their memory performance.

Sample characteristics are displayed in Table 1. Whereas there was no age difference in years of education, *t*(83) = 1.50, *p* = 0.14, when compared to young adults, older adults scored higher on the Shipley Vocabulary test, *t* = 4.32, *p* < 0.001, as well as had lower levels of depression, *t*(83) = 3.87, *p* < 0.001, anxiety, *t*(83) = 3.89, *p* < 0.001, and stress, *t*(83) = 4.11, *p* < 0.001. On the BIS/BAS, there were no age differences in drive, *t*(82) = 1.77, *p* = 0.08, fun seeking, *t*(83) = 1.86, *p* = 0.07, reward responsiveness, *t*(83) = −0.59, *p* = 0.56, and behavioral inhibition, *t*(59) = 0.39, *p* = 0.70.

#### Procedure

Experiment 4 used the same materials and protocol as Experiment 3—the only changes were made to the task instructions for the reward block of the directed forgetting task. After reading encoding instructions for this block, participants completed two multiple choice comprehension questions. In the first question, a sample trial was presented in which a $0.01 reward was paired with a TBR word. Participants were asked to indicate what the outcome would be if they remembered the TBR word from the following options: “You would win $0.01,” “You would lose $0.01,” or “You would not receive anything.” In the second question, the sample trial presented a $0.75 reward paired with a TBF word and participants selected what the outcome would be if they forgot the TBF word from the options: “You would win $0.75,” “You would lose $0.75,” or “You would not receive anything.” To ensure participants understood the retrieval instructions and the financial penalty for a committing a false alarm, they were asked to indicate what the outcome would be if they incorrectly identify a NEW word as one that they previously studied from the following options: “You will lose $0.50,” “You will win $0.50,” or “Nothing will happen.” If participants answered any of the questions incorrectly, the survey presented the correct answer and reiterated the instructions.

### Results

With regard to our comprehension check questions, nine older adults responded incorrectly to the TBF trial—three indicated they would lose $0.75 and six indicated they would receive no reward if they forgot the word. Two older adults also responded incorrectly to the TBR trial, indicating they would receive no reward if they remembered the word. Seven young adults incorrectly responded to the TBF trial—one indicated they would lose $0.75, and six indicated they would receive no reward. Regarding false alarm instructions, 10 older adults and eight younger adults incorrectly indicated that nothing would happen to their earnings for committing a false alarm, and five older adults and six younger adults indicated they would win $0.50.

**FIGURE 4:**
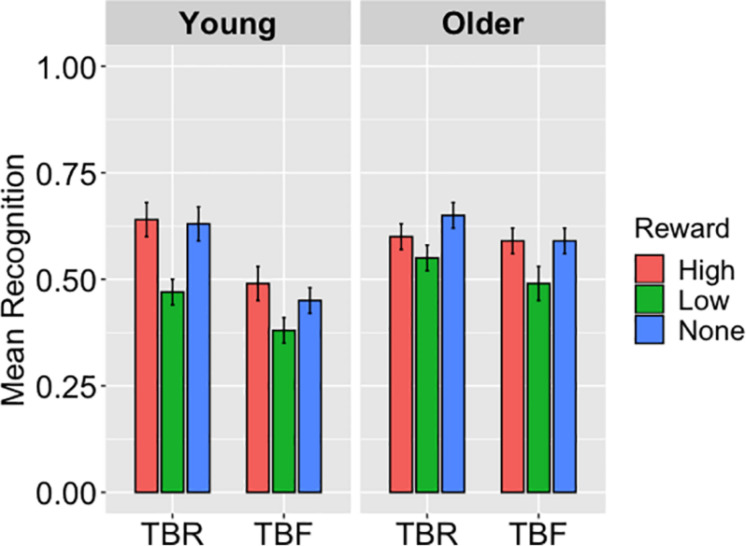
The average hits for to-be-remembered (TBR) and to-be-forgotten (TBF) words in Experiment 4, displayed as a function of reward magnitude and age group, illustrate a main effect of cue (*p* < 0.001) with better recognition of TBR than TBF words as well as an effect of reward (*ps* < 0.001), such that low-reward words were recognized to a lesser extent than both high- and no-reward words. An age-by-cue interaction is also displayed, as older adults recognized more TBF words than young adults (*p* = 0.01), but the same proportion of TBR words.

Recognition performance is displayed in Figure 4. Average hit rates were submitted to a 2 (Age Group: Young, Older) × 3 (Reward: High, Low, None) × 2 (Cue: TBR, TBF) repeated-measures ANOVA, which showed a main effect of reward, *F*(2,166) = 25.73, *p* < 0.001, η_p_^2^ = 0.24. According to pairwise comparisons, low-reward words (*M* = 0.47, *SD* = 0.21) were recognized to a lesser extent than high-reward (*M* = 0.58, *SD* = 0.19; *p* < 0.001) and no-reward words (*M* = 0.58, *SD* = *0.21*; *p* < 0.001). There was also a main effect of cue, *F*(1,83) = 32.07, *p* < 0.001, η_p_^2^ = 0.28, with better recognition of TBR (*M* = 0.59, *SD* = 0.19) than TBF words (*M* = 0.50, *SD* = 0.21). The main effect of age was not significant, *F* = 2.77, *p* = 0.10.

Replicating Experiment 3, there was a significant age group-by-cue interaction, *F*(1,83) = 9.31, *p* = 0.003, η_p_^2^ = 0.10. Pairwise comparisons showed that older adults recognized more TBF words (*M* = 0.55, *SD* = 0.20) than young adults (*M* = 0.44, *SD* = 0.20; *p* = 0.01), but a similar proportion of TBR words (*p* = 0.70), implying that older adults were less able to intentionally forget TBF words than young adults. There were no other significant interactions, *Fs* ≤ 2.77, *ps* ≥ 0.10.

The comparison of false alarm rates across age groups showed that there was no difference between young (*M* = 0.29, *SD* = 0.27) and older adults (*M* = *0.27*, *SD* = 0.18), *t* = 0.49, *p* = 0.61.

The proportion of words recalled was analyzed in a 2 (Age Group: Young, Older) × 3 (Reward: High, Low, None) × 2 (Cue: TBR, TBF) ANOVA. Like Experiment 3, overall recall was low (*M* = 0.05, *SD* = 0.04). The ANOVA revealed a main effect of reward, *F*(2,166) = 4.75, *p* = 0.01. Pairwise comparisons confirmed that recall was better for high-reward words (*M* = *0.07*, *SD* = 0.09) than for no-reward words (*M* = 0.04, *SD* = 0.06), *p* = 0.04; there was no difference in recall between high- and low-reward words (*M* = 0.04, *SD* = 0.05), *p* = 0.08, nor between low-reward and no-reward words, *p* = 1.00.

### Discussion

The results of Experiment 4 replicate those of Experiment 3, revealing the typically reported age difference in directed forgetting. Along with prior work (e.g., Titz and Verhaeghen, 2010), these results imply that older adults are less able to control their memory to intentionally forget TBF words. Further like Experiment 3, high rewards enhanced memory relative to low-reward items, but there was no difference in memory for high- and no-reward items. This effect of reward magnitude also did not vary based on memory instruction, suggesting that rewards had a general effect on memory performance as opposed to a differential effect on remembering and intentional forgetting.

The novel component of this experiment was the addition of comprehension checks, in which we probed whether participants understood how rewards would be administered based on performance (i.e., that they would win a reward for remembering TBR words as well as for forgetting TBF words and be penalized for committing a false alarm). Only 20% of older adults and 17.5% of young adults incorrectly answered these questions about encoding, and 33% of younger and older adults incorrectly answered the question at retrieval, all of whom were required to reread the instructions prior to beginning the experiment. Given that our results were consistent with Experiment 3, it does not seem likely that the effect of our reward manipulation (or lack thereof) on intentional forgetting can be attributed to misunderstanding instructions.

## General Discussion

Prior in-lab experiments have established that age is associated with decreased abilities to intentionally forget (Zacks et al., 1996; Titz and Verhaeghen, 2010). Across four experiments, we assessed the directed forgetting effect in an online sample of younger and older adults recruited from CloudResearch/Turk Prime with the main objective of elucidating whether reward anticipation could positively impact older adults’ reduced ability to intentionally forget.

This is the first study to establish a directed forgetting effect in an online sample. In all four experiments, we replicated the typical directed forgetting effect of better recognition memory for TBR words than for TBF words. The cognitive and neural mechanisms responsible for directed forgetting are still debated (Anderson and Hanslmayr, 2014; Aguirre et al., 2017), but one hypothesis is that participants simply do not search their memory as long for TBF words or choose to withhold retrieved information. It was unclear from the results of Experiment 2 when participants were rewarded for successful intentional remembering and forgetting, whether participants were withholding their memory or suppressing retrieval to increase their performance-based rewards. Replicating findings from Macleod (1999), in Experiments 3 and 4, we found little evidence of this as participants did not freely recall very many words overall, but recalled more TBR words, despite an added monetary bonus to recall TBF words. We chose to probe memory for additional TBF items with a free recall task instead of a recognition task to avoid source confusion. Results from a second recognition task would be unclear because it would not be able to tease apart memory for words that were encoded in the original encoding session and those encoded during the first recognition test. Recall is a cognitively harder task, especially for older adults, so some TBF items may have been missed by employing this method, but it avoids the confounds of a second recognition task.

Only in Experiments 3 and 4 did we find typical age-related reductions in directed forgetting. There were few age differences or interactions overall across the experiments, including no main effects of age. As crowdsourcing platforms like MTurk and CloudResearch/Turk Prime are utilized more often in psychological research, findings that have been well-documented in the lab may not replicate in online samples due to systematic differences between these samples (Paolacci and Chandler, 2014). Interestingly, our results suggest that this may not be because of differences in older adults who participate in-lab versus online, but because of younger adults. The age-related reductions in directed forgetting that we found in Experiments 3 and 4 seem to be driven by the amount of task engagement by younger adults. In online studies, younger adults are known to divide their attention between the task and other distractions such as their phone or television (Clifford and Jerit, 2014). In Experiments 3 and 4, when a detection task was included to increase engagement during encoding, younger adults showed a stronger directed forgetting effect than older adults, but both groups recognized TBR stimuli to the same extent. These findings suggest that online data collection might require that younger adult participants be given a more engaging encoding task that prevents divided attention, but not so cognitively demanding so as to decrease overall performance.

Turning to our main objective, in Experiments 2–4, we included a reward manipulation to determine whether added motivation might help older adults’ ability to intentionally forget by increasing cognitive control and goal-directed remembering *and* forgetting, or whether rewards may potentially hinder directed forgetting because of processes that unfold during reward anticipation that prioritize high value information in memory rather automatically, thereby making any stimuli associated with a high reward value during encoding more likely to be remembered. We found evidence across experiments that high-value reward anticipation boosted recognition memory for both younger and older adults compared to low-reward (Experiments 2–4) and compared to no-reward (Experiment 2), regardless of the memory cue to remember or forget. In other words, this evidence supports the latter hypothesis that reward anticipation increases the ability to encode and remember information but does not seem to help with intentional forgetting abilities. It has been suggested that age-related declines in cognitive control are responsible for the inability to inhibit unwanted information, and this leads to continued encoding of items they have been instructed to forget (Sahakyan et al., 2008; Gallant et al., 2018). Despite evidence that reward anticipation can improve cognitive control abilities in other tasks (Ferdinand and Czernochowski, 2018), our findings do not support that this is occurring in this paradigm. We found no evidence that high reward led to better intentional forgetting compared to low or no reward, ergo reward anticipation did not increase cognitive control abilities in the task for younger or older adults.

Instead, evidence from this set of studies was generally in support of our preregistered hypothesis (see footnote 1) that high-value reward anticipation boosts overall remembering and does not lead to increased goal-directed forgetting, but this was true for both younger and older adults. It is important to note that in Experiment 2, high-reward words were better recognized than low-reward and no-reward words which we had additionally hypothesized, but in Experiments 3 and 4, hit rates for high-reward did not differ from no-reward words. These differences in results may be accounted for by the change in encoding conditions with participants being more engaged (particularly younger adults) during Experiments 3 and 4 than in Experiment 2. Although speculative, this increased engagement may be coupled with psychological differences between the two experimental blocks. In block 1, there was no reward manipulation and participants were unaware that the next block of trials would include performance-based rewards. This may have led to more cognitive resources available to encode the stimuli in the no-reward block of Experiments 3 and 4 since interference from other trials is low at this early stage of the task. In block 2, when cognitive resources become limited due to processing the reward cue and stimulus simultaneously, as well as the sheer number of trials that have occurred at that point, participants may expend more cognitive effort on high-reward compared to low-reward trials, leading to no statistical differences in recognition memory for high- and no-reward trials. We intentionally did not counterbalance the no-reward/reward blocks to ensure that knowledge of monetary incentives did not influence performance on the no-reward block. Future studies that are able to counterbalance block order, or that include the same number of trials, but all associated with rewards, will be able to test the idea of these psychological differences and the role of interference on this pattern of results.

An additional difference between the blocks is that the reward cue appears on the screen during stimulus presentation during block 2. The purpose of presenting the reward cue during stimulus presentation was to test the effect of reward anticipation on the ability to control memory by either intentionally remembering or forgetting the word. Reward anticipation has been shown to engage the reward network but also other brain regions that could either increase inhibitory cognitive control of memory that would benefit both goal-directed remembering and forgetting (e.g., Cohen et al., 2016; Ferdinand and Czernochowski, 2018) or more automatic episodic memory formation (e.g., Spaniol et al., 2014; Cohen et al., 2019; Bowen et al., 2020) that would benefit remembering only, making forgetting more difficult. This is the first study to examine the role of monetary reward anticipation and its interaction with memory cues in a directed forgetting paradigm, adding to a small literature examining the effect of motivation on directed forgetting, more broadly. In an early study, Macleod (1999) found that monetary reward during a surprise free recall test did not elicit additional TBF words from memory. Utilizing points as a proxy for remember and forget cues presented after stimulus encoding, Friedman and Castel (2011) found a stronger directed forgetting effect when participants were motivated by these points compared to baseline. Finally, presenting reward cues along with memory cues after stimulus presentation led to increased directed forgetting when the rewards were gains, but a reduced ability to forget when participants were expecting a loss(Ren et al., 2018). There are still many unanswered behavioral questions, such as whether the timing of the reward cue matters to intentional forgetting processes (i.e., cueing rewards before, during, or after stimulus encoding or cueing during retrieval). Perhaps reward anticipation during stimulus encoding in the current study reduced the ability for older adults to cognitively control their memory because it was too cognitively taxing to read the stimulus, pay attention to the reward cue, and then engage with the TRB or TBF cue. Future studies that manipulate this aspect of the experimental design will be able to answer these questions and align the findings with other studies that have examined the role of reward on directed forgetting. Further, and of importance for elucidating the role of cognitive control, individual differences in older adults’ executive function may also be a predictor of reward effects on directed forgetting. As noted, we did not find evidence that reward was leading to increased cognitive control in this paradigm when participants were put into a state of reward anticipation during stimulus encoding, but it is still unclear from this set of studies how reward is influencing these different effects. An interesting future experiment would be to use neuroimaging to further clarify the role of prefrontal cortex and cognitive control regions, reward network activation and dopaminergic modulation of hippocampal consolidation processes (e.g., Adcock et al., 2006; Spaniol et al., 2014; Bowen et al., 2020), and/or left lateral prefrontal cortex engagement indicative of increased semantic processing of the verbal stimuli at the time of encoding (Cohen et al., 2016) to test how each supports the relationship between reward anticipation and directed remembering and forgetting.

## Conclusion

In four experiments, we tested directed remembering and forgetting abilities in an online CloudResearch/Turk Prime sample of younger and older adults. We replicated typical age-related deficits in forgetting, but only when younger adults were obliged (via button press) to stay cognitively engaged in the task. This highlights the importance of task demands in online studies—not only to be mindful of cognitive limitations of older adults but also to prevent possible divided attention in younger adult samples. In line with our preregistered hypotheses, across three experiments, we found evidence that high-value reward anticipation led to better memory overall compared to low reward, for younger and older adults, but this was regardless of the directed forgetting cue. If reward anticipation increased cognitive control in this paradigm, this would have modulated the directed forgetting effect, not general memory overall. High-value reward anticipation may strengthen memory relatively automatically, rather than strategically, possibly via dopaminergic activation of memory formation processes. Moreover, bonus rewards for successful recall of TBF words revealed that participants were not strategically withholding their memory of TBF words in the service of a higher payout, giving strength to the idea that directed forgetting effects are not driven by a motivational retrieval strategy, but by processes that unfold at the time of encoding. Future studies aimed at uncovering the cognitive and neural mechanisms responsible for these effects will be necessary to understand how these processes remain relatively stable across the life span.

## Data Availability Statement

The datasets generated for this study are available on the Open Science Framework https://osf.io/3pe9d/.

## Ethics Statement

The studies involving human participants were reviewed and approved by the Southern Methodist University Institutional Review Board. The patients/participants provided their written informed consent to participate in this study.

## Author Contributions

HB and SG contributed to the conception and design of the study. SG programmed the study, organized the online data collection, maintained the participant data, and performed the statistical analysis. DM performed the statistical analyses. HB and SG wrote the first draft of the manuscript. All the authors wrote sections of the manuscript, contributed to manuscript revision, read and approved the submitted version.

## Conflict of Interest

The authors declare that the research was conducted in the absence of any commercial or financial relationships that could be construed as a potential conflict of interest.

## Footnotes

1 https://osf.io/3pe9d/
